# Supplementary material for: Cell cycle-coupled CK1δ turnover, autoinhibition, and activity
Source: eLife. 2026 Oct 28;15:RP112381. doi: 10.7554/eLife.112381 (PMC13716387; doi:10.7554/eLife.112381)
Supplement: Supplementary file 4 [file elife-112381-fig5-data1.docx]

**Figure 5B – Source Data 1.** Left: Original film corresponding to Figure 5B. CK1δ-FLAG was induced followed by a double thymidine block and release and sampled as shown on Figure 5A. Resulting blots were decorated with anti-FLAG antibody. Right: Original Ponceau Staining scan corresponding to Figure 5B. n = 3.

**Figure 5D – Source Data 1.** Left: Original film corresponding to Figure 5D. CK1δ-FLAG was induced followed by a cell cycle arrest protocol and sampled as shown on Figure 5C. Resulting blots were decorated with anti-FLAG antibody. Right: Original Ponceau Staining scan corresponding to Figure 5D. n = 3.

**Figure 5E – Source Data 1.** Left: Original film corresponding to Figure 5E. U2OStx cells were treated with a cell cycle arrest protocol and sampled as shown on Figure 5C. Resulting blots were decorated with anti-CK1δ antibody. Right: Original Ponceau Staining scan corresponding to Figure 5E. n = 3.
