# Supplementary material for: Cell cycle-coupled CK1δ turnover, autoinhibition, and activity
Source: eLife. 2026 Oct 28;15:RP112381. doi: 10.7554/eLife.112381 (PMC13716387; doi:10.7554/eLife.112381)
Supplement: Peer review history

# Editor report: eLife Assessment

**DOI:** 10.7554/eLife.112381.3.sa0

**Authors:** Volker Dötsch

**Affiliations:** Goethe University Frankfurt Germany

**Keywords:** Incomplete, Valuable

This manuscript presents **valuable** experimental results describing the localisation and regulation of casein kinase CK1δ during the cell cycle. During the G2 phase of the cell cycle, the autophosphorylation of the C-terminal tail stabilises and inhibits the kinase which might protect CK1δ in the subsequent G1 phase. The results may be of interest to researchers working on casein kinase function and regulation but due to lack of some controls remain **incomplete**.

[Editors' note: this paper was reviewed by Review Commons.]

# Referee report: Reviewer #1 (Public review)

**DOI:** 10.7554/eLife.112381.3.sa1

Summary:

Involved in various cellular pathways and processes, Ser/Thr kinase CK1δ is thought to be constitutively active and its tail autophosphorylation is suggested as a putative inhibitory mechanism of CK1δ kinase activity. Here, the authors investigate CK1δ's phosphorylation status, in relation to its location and dynamics throughout the cell cycle. Immunofluorescence and biochemistry studies showed that the subcellular distribution of CK1δ is dynamic and in equilibrium between centrosomal and nuclear pools. The authors argue that CK1δ phosphorylation protects it from degradation. Finally, the authors show differences in CK1δ phosphorylation status and location throughout the cell cycle. Combining their findings with the existing literature, they propose a model of CK1δ phosphorylation status, location and dynamics throughout the cell cycle.

Comments on latest version:

The authors have unfortunately taken a very defensive mode There are two themes that authors resort to: (1) We have published previously with some reagents - therefore these are well-established and we do not need to include controls anymore; (2) Independent biological replicates and statistics are only appropriate in some places, again because certain observations have been previously published (by the same authors) and well-established. In my opinion, this is not how rigorous research should be done. Specially with IF, it is essential in every experiment to have robust negative controls.

# Referee report: Reviewer #2 (Public review)

**DOI:** 10.7554/eLife.112381.3.sa2

Summary:

In this study, Serrano et al. employed a combination of cell biological and molecular approaches to investigate the localization and regulation of Casein Kinase CK1 during the cell cycle using U20S cells. They show that CK1 dynamically localizes between the centrosomes and the nucleus but can be sequestered away from the centrosomes upon overexpression of its binding partner PER2. They provide evidence that CK1 WT but not a phospho-null mutant strongly accumulates in a hyperphosphorylated form upon inhibition of phosphatases (using Calyculin and Okadaic Acid) and thus conclude that CK1 tail phosphorylation protects the kinase from degradation. Using synchronized cells, they show that CK1 accumulates unphosphorylated in S-phase (APC/Cdh1 inactive) but phosphorylated at the G2-M transition. Immunostaining shows that CK1 localizes to the centrosomes during mitosis.

Strengths:

The authors reveal that the activity and abundance of dephosphorylated and phosphorylated CK1δ are regulated in a cell cycle-dependent manner. This suggests that these different pools are associated with distinct physiological functions.

Comments on latest version:

Unfortunately, problems with controls remain in the revised manuscript. At the very least, the authors should include all the necessary controls in their study instead of referring to previously published papers. I have asked for a detailed description of the kinase construct in which the phospho-Serine and Threonine of the C-terminal tail are substituted, but I did not see any more information in the revised version.

# Author comment: Author response

**DOI:** 10.7554/eLife.112381.3.sa3

**Authors:** Fidel Emmanuel Serrano, Bianca Ruppert, Axel CR Diernfellner, Michael Brunner

**Affiliations:** Heidelberg University Heidelberg Germany; Heidelberg University Heidelberg Germany; Heidelberg University Heidelberg Germany; Heidelberg University Heidelberg Germany

The following is the authors’ response to the original reviews

**Public Reviews:**

**Reviewer #1 (Public review):**

We appreciate the authors have provided answers to many of the points we raised, and the changes made to their manuscript, which we think strengthen the overall evidence presented. However, we find that some important controls are still missing across experiments.

Major comments:

(1) Shortcomings in Immunofluorescence experiments:

a. Antibody cross-reactivity was only tested against CK1ɛ, but should also be tested against CK1α, which is abundant in U2OS cells, and is also known to be involved in cell-cycle regulation.

We selected the CK1δ antibody (abcam, ab85320 clone AF12G4) precisely because of its well-established specificity for CK1δ. In many well-cited studies [An et al., Nature Communications, 2022, PMID: 35810166], [Eng et al., PLOS One 2017, PMID: 28545154], some of which involve direct comparison with CK1α and CK1ε [Sinnberg et al., BMC Cancer, 2016, PMID: 27488834], [Beale et al., Journal of Biological Rhythms, 2019, PMID: 30898060], the CK1δ (ab85320) antibody is cited directly as the antibody used to specifically detect CK1δ.

Additionally, through our previous eLife paper, we have confirmed the observation from previous studies that clearly demonstrate that this CK1δ (ab85320) antibody can specifically recognize CK1δ and not even its closest homolog CK1ε. Given the high degree of conservation in the kinase region between CK1δ and CK1ε, this implies that this antibody is likely directed at the divergent C-terminal tail (see sequence alignments in Author response image 1). Given that CK1α C-terminal tail is even more divergent from CK1δ, along with its reliable use in the literature from multiple working groups, it is safe to assume that this antibody can reliably distinguish CK1δ from CK1α.

**Author response image 1.** Protein sequence alignments between CK1δ, CK1ε, and CK1α highlighting high sequence conservation in the structured kinase domain and sequence divergence at the C-terminal tail.

b. Fig. 1: Statistical analyses are missing from the analysis.

We have clearly added statistical analysis showing significant differences of CK1δ localization at the centrosome.

c. Fig. 2: No colocalisation analysis shown for figure 2, only some arrowheads pointing to puncta. Appropriate colocalisation statistics are important since for practical reasons, only a few representative images can be shown on the figure.

CK1δ colocalization is already well-established in the literature and additionally demonstrated in this study with the clear colocalization in Figures 1C and 1D. In Figure 2, we have chosen to report representative images which correspond to the typical phenotype we observe and can compare side-by-side which show clear and striking differences in terms of CK1δ localization between (a) cells that form foci and lose CK1δ localization at a clear and distinct centrosome and (b) cells that do not form foci and CK1δ is clearly localized to the apparent centrosome. By showing several fields-of-view, we demonstrate that this is the prevalent comparative phenotype.

d. Fig. 6: Even if the figure is illustrative, it is important to show centrosome staining to visualise CK1ẟ's recruitment to the centrosome in G2/prophase, especially since this information is used to propose the model in figure 7.

It is well-established that centrosomal duplication is a key hallmark of G2. Combined with our observations from Figure 2 showing that CK1δ to distinct puncta that one can only assume is the centrosome, and now showing in Figure 6, a clear duplication of these puncta. Importantly, this occurs in cells that also exhibit higher DAPI staining consistent with the duplicated and condensed genomic state when compared to other cells in the field of view, another hallmark of G2. Therefore, additional centrosomal staining and colocalization will only confirm what we already know about typical hallmarks of G2 and wider mitosis and the established CK1δ localization to the centrosomes.

e. For all figures: Please mention the number of independent biological replicates in the figure legends (1, 2, 6). For figure 1, if there are 3 independent biological replicates, the quantification should take all of them into account (as opposed to the data points corresponding to 10 cells), and statistics must be done appropriately, taking those independent replicates into account. Same for the colocalisation analysis in figure 2 once you include it.

We have mentioned the number of independent biological replicates where we considered it appropriate in Figures 3, 4, and 5. Figure 1 A and B are representative fields-of-view which show a replicable phenotype and we have quantified 10 cells to show the statistical difference in CKδ colocalization to PCNT. Figure 1 C and D show individual representative cells used for the line plot analysis but as one can already see from Figures 1 and B, these observations are clearly reproducible. Figure 2 A is an additional representative photo from observations we have made in our previous eLife publication. Figure 2 B already shows multiple fields-of-view which show a replicable result. Figure 6, again, shows a representative series of cells which already clearly demonstrate the change in CK1δ localization over the course of mitosis.

(2) Shortcomings in biochemistry experiments:

On CalA control, this is not a matter of confirming that CalA treatment works in principle, but rather to confirm that CalA treatment worked in this specific replicate. Aliquots may lose potency (e.g. with freeze-thaw cycles / exposure to light), hence checking for enrichment of phospho-proteins is essential to confirm the treatment was successful in this particular instance. In the worst-case scenario, the company may have sent the wrong compound altogether! A positive and a negative control is the basis for every experiment to make meaningful interpretation. On a separate note, many experiments have control and siRNA or compound treatments on two different gels - this should be rectified as they are meaningless if different exposures have been selected for different immunoblots.

I find it hard to seriously answer to these points. I do not know what kind of reagents the reviewer is used to work with. On every experiment we show as a sub-figure, we use the same CalA that clearly works as intended since we can clearly see a phosphorylation-induced gel shift of the kinase. This rules out aliquots losing their potency or supplier providing the wrong compound. We also show a clear negative control in showing the absence of the gel shift prior to CalA treatment.

On a separate note, we have no experiments with siRNA so we are unsure what the reviewer is referring to here. Of the 11 immunoblots we report here, each of which are representative of a triple replicate, only 1, Figure 3B, shows a split view on an equal exposure. Even then this was actually run on the same gel and this figure was simply spliced to simplify the message we want to convey. Therefore, we believe we have used the appropriate controls and level of rigor to make our observations and interpret them adequately.

(4) As the authors mention, the kinase is not fully inactive when tail phosphorylated. Recent research has also suggested that tail-phosphorylated CK1ẟ may show increased catalytic activity for a few select, specific substrates, in the co-occurrence of pT220 (Cullati et al., 2022; Cullati et al., 2024). It is thus tricky to directly infer that phosphorylated CK1ẟ is inhibited, when no positive control for CK1ẟ inhibition was shown in the evidence presented. It would be necessary to either nuance your claim or include a positive control for CK1ẟ inhibition. Please revise statements in the manuscript accordingly.

We have clearly demonstrated that CKδ tail phosphorylation leads to kinase inhibition and refer the reviewer to our work in PNAS [Marzoll et al., PNAS, 2021; PMID: 35217617].

(5) It would be important to include statistical analyses for the immunofluorescence data in Fig. 1 and 2.

We have included statistical analysis where we considered it appropriate in Figure 1 A and B. In Figure 1 C and D, statistical analysis is not appropriate to apply to compare single line plots as the individual data are clearly distinct from one another. For Figure 2 A and B, again, we are showing this data to show what is already a clear difference in CK1δ centrosomal localization when we compare cells that either form PER-CRY foci or do not.

(9) The authors mentioned "In the eLife study, we show that inhibition of kinase activity by PF670462 stabilizes CK1δ and that the overexpressed kinase-dead mutant CK1δ-K38R is stable." Unfortunately, the data from biochemical analyses presented in the eLife publication is uninterpretable due to a lack of loading controls.

We respect the reviewer’s opinion on this matter. However, the earlier study was independently peer-reviewed and has already been published. The reviewer was not involved in the assessment of that work, and we therefore feel that a retrospective evaluation of its merits is beyond the scope of the present review. We would respectfully ask that the current manuscript be evaluated on the basis of the questions it addresses and the evidence presented here.

(10) While the data presented in Penas et al. strongly suggests a link between CK1ẟ stabilisation and the APC/C-Cdh1 complex, it is the only study to have shown it. Given that (1) science relies on data reproducibility and (2) your proposed model relies heavily on the relationship between CK1ẟ stabilisation and the APC/CCdh1 complex, it would be appropriate to include the investigations mentioned in our original comment.

We respectfully point out that the purpose of the present review is to assess the manuscript under consideration, not to retrospectively reassess data that have already been published, such as those reported by Penas et al., even if this is the only study we cite in support of this particular point.

After checking the peer review received from Review Commons, we cannot see the “investigations in our original comment” that this reviewer is referring to.

**Reviewer #2 (Public review):**

In this study, Serrano et al. employed a combination of cell biological and molecular approaches to investigate the localization and regulation of Casein Kinase CK1 during the cell cycle using U20S cells. They show that CK1 dynamically localizes between the centrosomes and the nucleus but can be sequestered away from the centrosomes upon overexpression of its binding partner PER2. They provide evidence that CK1 WT but not a phospho-null mutant strongly accumulates in a hyperphosphorylated form upon inhibition of phosphatases (using Calyculin and Okadaic Acid) and thus conclude that CK1 tail phosphorylation protects the kinase from degradation. Using synchronized cells, they show that CK1 accumulates unphosphorylated in S-phase (APC/Cdh1 inactive) but phosphorylated at the G2-M transition. Immunostaining shows that CK1 localizes to the centrosomes during mitosis.

The manuscript has improved overall, but some sections are still inconclusive and require clarification.

Major comments:

Figure 1 is inconclusive. CK1 nuclear staining is highly similar in untreated cells and in cells treated with CHX + PF670462. The reduction in centrosomal staining in these cells is barely significant. However, the authors draw very strong conclusions from these data sets. In panel B, the cells appear to have been fixed incorrectly, and the anti-PCNT shows a strong background signal. Not convinced that immunofluorescence is the best approach to look at protein dynamics in vivo.

Figures 1 A and B are meant to show how CHX and PF670462 may affect CK1δ localization to the centrosome (stained by PCNT) over several cells whereas Figures 1 C and D are meant to more clearly show this effect in individual cells. This effect is indeed modest in the endogenous situation compared to the overexpressed which shows a clearer redistribution of CK1δ. We show this when we compare the quantification in Figure 1A and the colocalization analysis in Figure 1 C (right panel; showing that some CK1δ still colocalizes to the PCNT-stained centrosome) to their overexpressed counterparts. This however, only emphasizes the strength of our overexpression approach as it has allowed us to observe a more dramatic shift in CK1δ centrosomal localization.

In Figure 2, panel B, the authors should co-stain the centrosomes of cells that co-express CRY1 and CK1, as some of these dots may represent the centrosomes.

In principle, we indeed cannot exclude the possibility that a CRY-stained foci may actually be a centrosome. However, we refer to our comments to Reviewer #1 and add our observation that these PER-CRY foci are clearly nuclear based on our earlier eLife publication and are therefore unlikely to be centrosomal. (If some of the dots represent centrosomes, this would mean that a fraction of CRY1 is recruited from the nucleus to the centrosome in the cytosol. There is no mechanism for this known.)

Figures 3B, please provide information on the non-phosphorylable CK1a mutant (it is mentioned as a variant in which all serine and threonine residues in the C-terminal tail were replaced by alanine). Specify the number of sites mutated and their exact position. Is this non-phosphorylable CK1a version catalytically active?

As said, all serine and threonine residues in the tail have been changed, and yes, the kinase is of course active.

Treatment of samples with inactivated PPase should be used as a control.

It is unclear what this experiment would control for and how its outcome would affect any of the conclusions of the present study.

Strengths:

The authors reveal that the activity and abundance of dephosphorylated and phosphorylated CK1δ are regulated in a cell cycle-dependent manner. This suggests that these different pools are associated with distinct physiological functions.

Weaknesses:

Unfortunately, some of the data are inconclusive, and there is no data/information linking the cell cycle regulation of CK1δ to its function during the cell cycle.

Although CK1δ continuously undergoes cycles of autophosphorylation and dephosphorylation, the kinase is predominantly found in its dephosphorylated, active state under steady-state conditions. This has obscured the physiological relevance of its well-established inhibitory autophosphorylation. We now identify mitosis as the cellular state in which this equilibrium shifts: autophosphorylated and consequently inhibited CK1δ accumulates. These findings resolve a longstanding question regarding the physiological function of CK1δ autophosphorylation and reveal it as a cell-cycle-regulated mechanism of kinase inhibition.

**Recommendations for the authors:**

**Reviewer #1 (Recommendations for the authors):**

Most of our minor comments related to editorial issues have been ignored by the authors and the errors persist. Simple corrections will improve the manuscript and make it more readable.

We also found new minor errors as follows:

69-70: 'dependent on its kinase is activity' --> 'its kinase activity'

164: 'sites or targeted by' --> 'sites are targeted by'

179: 'PF670' instead of 'PF670462'

292: 'against higher the steady state levels' --> 'against the higher steady state levels' or 'against higher steady state levels'

We thank Reviewer #1 for their close reading of our text. We have adjusted the text accordingly.
